# Supplementary material for: Resolving the full spectrum of human genome variation using Linked-Reads
Source: Genome Res. 2019 Apr;29(4):635–45. doi: 10.1101/gr.234443.118 (PMC6442396; doi:10.1101/gr.234443.118)
Supplement: Supplemental Material [file supp_gr.234443.118_Supplemental_longranger-2.2.zip › longranger-2.2.tar.bz2!/lr2.2-public/lib/stan/cmdstan/stan/doc/api/html/agrad_2fwd_2functions_2multiply__log_8hpp_source.html]

Stan: src/stan/agrad/fwd/functions/multiply\_log.hpp Source File


|  |  |
| --- | --- |
|  | Stan  2.5.0  probability, sampling & optimization |


- Main Page
- Related Pages
- Namespaces
- Classes
- Files

- File List
- File Members

All Classes Namespaces Files Functions Variables Typedefs Enumerations Enumerator Friends Macros Pages

- src
- stan
- agrad
- fwd
- functions

multiply\_log.hpp

Go to the documentation of this file.

1 #ifndef STAN\_\_AGRAD\_\_FWD\_\_FUNCTIONS\_\_MULTIPLY\_LOG\_HPP

2 #define STAN\_\_AGRAD\_\_FWD\_\_FUNCTIONS\_\_MULTIPLY\_LOG\_HPP

3

4 #include <stan/agrad/fwd/fvar.hpp>

5 #include <stan/meta/traits.hpp>

6 #include <stan/math/functions/multiply\_log.hpp>

7

8 namespace stan {

9

10  namespace agrad {

11

12  template <typename T>

13  inline

14  fvar<T>

15  multiply\_log(const fvar<T>& x1, const fvar<T>& x2) {

16  using stan::math::multiply\_log;

17  using std::log;

18  return fvar<T>(multiply\_log(x1.val\_, x2.val\_),

19  x1.d\_ \* log(x2.val\_) + x1.val\_ \* x2.d\_ / x2.val\_);

20  }

21

22  template <typename T>

23  inline

24  fvar<T>

25  multiply\_log(const double x1, const fvar<T>& x2) {

26  using stan::math::multiply\_log;

27  using std::log;

28  return fvar<T>(multiply\_log(x1, x2.val\_),

29  x1 \* x2.d\_ / x2.val\_);

30  }

31

32  template <typename T>

33  inline

34  fvar<T>

35  multiply\_log(const fvar<T>& x1, const double x2) {

36  using stan::math::multiply\_log;

37  using std::log;

38  return fvar<T>(multiply\_log(x1.val\_, x2),

39  x1.d\_ \* log(x2));

40  }

41  }

42 }

43 #endif

multiply\_log.hpp

stan::math::multiply\_log

boost::math::tools::promote\_args< T\_a, T\_b >::type multiply\_log(const T\_a a, const T\_b b)

Calculated the value of the first argument times log of the second argument while behaving properly w...

**Definition:** multiply\_log.hpp:51

stan::agrad::fvar

**Definition:** fvar.hpp:13

traits.hpp

fvar.hpp

stan::agrad::multiply\_log

fvar< T > multiply\_log(const fvar< T > &x1, const fvar< T > &x2)

**Definition:** multiply\_log.hpp:15

stan::agrad::fvar::d\_

T d\_

**Definition:** fvar.hpp:16

stan::agrad::log

fvar< T > log(const fvar< T > &x)

**Definition:** log.hpp:15

stan::agrad::fvar::val\_

T val\_

**Definition:** fvar.hpp:15


---

[ Stan Home Page ]

*© 2011–2014,
Stan Development Team.*
